# First Clinical Case of Equine Parvovirus-Hepatitis-Related Theiler’s Disease in Asia

**DOI:** 10.3390/v13101917

**Published:** 2021-09-24

**Authors:** Jungho Yoon, Taemook Park, Ahram Kim, Jongyoung Park, Byung-Joo Park, Hee-Seop Ahn, Hyeon-Jeong Go, Dong-Hwi Kim, Soontag Jung, Yeeun Seo, Joong-Bok Lee, Seung-Yong Park, Chang-Seon Song, Sang-Won Lee, In-Soo Choi

**Affiliations:** 1 Equine Clinic, Jeju Stud Farm, Korea Racing Authority, Jeju 63346, Korea; junghoy11@gmail.com (J.Y.); taemook7@gmail.com (T.P.); aidia0207@naver.com (A.K.); penditis@gmail.com (J.P.); 2 Department of Infectious Diseases, College of Veterinary Medicine, Konkuk University, Seoul 05029, Korea; twilightsd@naver.com (B.-J.P.); heesuob2@naver.com (H.-S.A.); misilseju@naver.com (H.-J.G.); opeean0@naver.com (D.-H.K.); virus@konkuk.ac.kr (J.-B.L.); paseyo@konkuk.ac.kr (S.-Y.P.); songcs@konkuk.ac.kr (C.-S.S.); odssey@konkuk.ac.kr (S.-W.L.); 3 Department of Food and Nutrition, College of Biotechnology and Natural Resources, Chung-Ang University, Anseong 17546, Korea; amazing2257@gmail.com (S.J.); sweet970@naver.com (Y.S.)

**Keywords:** equine parvovirus, horses, Theiler’s disease, hepatitis, virus shedding, in situ hybridization

## Abstract

Equine parvovirus-hepatitis (EqPV-H) is a newly identified etiologic agent of Theiler’s disease (TD). We present a case of EqPV-H-related fulminant hepatitis in a 14-year-old thoroughbred mare in Korea. The mare had acute hepatopathy and gastrointestinal symptoms, with abnormal liver-related blood parameters. The horse was born in the USA and imported to Korea in 2017, with no history of administration of equine biological products after entry into Korea. The horse was diagnosed with EqPV-H-associated hepatitis after abdominal ultrasonography, laparotomy, and nested polymerase chain reaction (PCR) and in situ hybridization (ISH) assays. The serum, nasal swab, oral swab, and liver biopsy were positive for EqPV-H according to the PCR assay. Genetic analysis of the partial NS1 gene of EqPV-H showed a unique nucleotide substitution, distinct from that in previously deposited strains. EqPV-H DNA was found not only in hepatocytes but also in bile duct epithelium and Kupffer cells, particularly via ISH. To the best of our knowledge, this is the first case of EqPV-H-associated TD in Asia, providing the first clinical evidence for viral shedding from the mouth and nose, and identification of EqPV-H in the liver. This study contributes to a better understanding of the pathological features of EqPV-H-associated TD.

## 1. Introduction

Theiler’s disease (TD), also known as equine serum hepatitis, is an important causative agent in fulminant hepatitis in horses [1]. TD transmission is usually associated with the inoculation of contaminated equine biological products such as tetanus antitoxin, and experimental oral transmission with viremic serum was recently demonstrated [2,3,4,5,6,7].

Since 2011, the following four viruses have been identified as candidate etiologic agents of TD using the advanced unbiased deep sequencing technique: equine hepacivirus (EqHV), equine pegivirus (EPgV), TD-associated virus (TDAV), and equine parvovirus-hepatitis (EqPV-H) [8,9,10,11]. However, after the discovery of EqPV-H in 2018, EqPV-H was detected in most clinical cases of TD [2]. Additionally, several consecutive studies conducted in the USA showed that 27 out of 28 (96.4%) of the clinical TD cases were positive for EqPV-H and the results experimentally demonstrated hepatotropism and the clinical correlation of EqPV-H with TD [3,4,5,6]. An original study sample of TDAV infection involved EqPV-H coinfection [11], and both EPgV and TDAV have been shown to be not hepatotropic and are not associated with hepatitis [12]. EqHV is related to the development of chronic hepatitis; however, reports of clinical cases of EqHV-associated TD are scarce, and only one severe hepatopathy case showed that EqHV caused hepatitis [2,13]. In view of this, EqPV-H is currently considered as a major etiologic agent of TD [2,4,11].

The clinical significance of EqPV-H in liver diseases varies from subclinical to acute severe hepatitis and liver failure [2,4]. A recent study also revealed that persistent infection with EqPV-H over five years caused chronic viremia in subclinical horses [14]. In surveillance studies conducted on clinically healthy horses in the USA, China, Germany, Austria, and Brazil, the prevalence of EqPV-H DNA ranged from 7.1% to 17%, and the seroprevalence ranged from 15% to 34.7% [2,11,15,16,17,18,19,20].

Despite the worldwide distribution and increasing importance of EqPV-H-associated TD in horses, clinical investigations and case reports on EqPV-H infections are limited; moreover, awareness about the potential risk of EqPV-H infection is relatively lower than awareness about other diseases among equine practitioners [2]. In this study, we present a case of EqPV-H-related acute hepatitis and liver failure that was diagnosed via a polymerase chain reaction (PCR) assay and in situ hybridization (ISH), using serum, nasal/oral swabs, and a liver biopsy.

## 2. Materials and Methods

### 2.1. Sample Collection

A 14-year-old thoroughbred mare was brought to the Jeju Stud Farm Equine Clinic of the Korea Racing Authority on 12 June 2021 with clinical signs of acute hepatitis and colic. Blood, nasal/oral swabs, and a liver sample were collected for diagnostic examination and exploratory laparotomy. Blood samples were drawn by venipuncture into three types (SST, K_2_ EDTA, and lithium heparin) of Vacutainer^®^ blood collection tubes (Becton Dickinson, Franklin Lakes, NJ, USA). Nasal/oral swabs were collected using sterile nylon swabs and immersed in a tube containing 2 mL of virus transport medium (Noble Bio, Hwaseong, Korea). Liver samples were collected by partial resection under sterile operation conditions. The resected liver sample was soaked in 10% neutral-buffered formalin or stored at −70 °C for pathological analysis and virus detection. Serum and swab samples were immediately used or kept at 4 °C for a complete blood cell count (CBC), serum biochemistry, and virus detection, and then stored at −70 °C pending further analysis. The animal protocols for this study were reviewed and approved by the Institutional Animal Care and Use Committee of Korea Racing Authority (KRA IACUC-2106-AEC-2106, 25 February 2021).

### 2.2. Virus Detection and Blood Analysis

Two causative viruses of clinical TD (EqPV-H and EqHV), together with the hepatitis E virus (HEV), which is another zoonotic pathogen of liver diseases to which equids are susceptible [21], were analyzed using the nested PCR technique [11,22,23,24]. Viral nucleic acids were extracted using a Patho Gene-Spin™ DNA/RNA Kit (Intron Biotechnology, Seongnam, Korea) from serum, oral and nasal swabs, and liver biopsy tissue, according to the manufacturer’s instructions. The PCR products were analyzed using 1.5% agarose gel electrophoresis and positive products were purified using a MEGAquick-spin Plus DNA Purification Kit (Intron Biotechnology, Korea) for subsequent commercial sequencing (Cosmo Genetech, Seoul, Korea). The partial NS1 gene of EqPV-H DNA confirmed in this study was submitted to GenBank (accession no. MZ923508). Additional PCR information and primer sets are listed in Supplementary Materials (Table S1).

The CBC and the biochemical analysis were performed using VetScan HM5 and VS2 analyzers, respectively (Abaxis, Union City, CA, USA). Blood lactate levels were generated using an Accutrend Plus analyzer (Roche, Mannheim, Germany).

### 2.3. Phylogenetic Tree Analysis

Molecular Evolutionary Genetics Analysis (MEGA) software, version X, was used to evaluate the phylogenetic tree of the identified partial EqPV-H sequence. Corresponding sequences were downloaded from the National Center for Biotechnology Information (https://www.ncbi.nlm.nih.gov/nucleotide/, accessed on 29 August 2021). Sequence alignment was performed using MUSCLE algorithm, and a phylogenetic tree was constructed using the maximum likelihood method (with a bootstrapping value of 500).

### 2.4. Histopathology

The surgical biopsy sample (1 × 1 × 1 cm) was fixed using a 10% neutral-buffered formalin solution (Sigma, St. Louis, MO, USA) overnight. A paraffin block of fixed tissue was prepared by standard tissue processing with graded ethanol and xylene. Formalin-fixed paraffin-embedded (FFPE) tissue sections of 4 μm thickness were prepared on a SuperFrost slide (Fisher Scientific, Pittsburgh, PA, USA) and stained with hematoxylin and eosin [25].

### 2.5. In Situ Hybridization

A digoxigenin-labeled DNA probe was prepared according to the process described in previous publications [25,26]. The nested PCR product was amplified with the following primers: 5′-GGA GAA GAG CGC AAC AAA TGC A-3′ (external forward), 5′-AAG ACA TTT CCG GCC GTG AC-3′ (external reverse), 5′-GCG CAA CAA ATG CAG CGG TTC GA-3′ (internal forward), and 5′-GGC CGT GAC GAC GGT GAT ATC-3′ (internal reverse) [11]. The presence of the 435-bp PCR product was confirmed using 1% agarose gel electrophoresis and the product was purified using a Wizard^®^ SV Gel and PCR Clean-Up System (Promega, Madison, WA, USA). The purified DNA was labeled using a digoxigenin DNA labeling kit (Roche, Basel, Switzerland) according to the standard protocol.

To detect EqPV-H DNA in the liver tissue, in situ hybridization was performed by following a previous protocol [26]. Briefly, rehydrated FFPE tissue sections were treated with 0.2 N HCl, and the treated sections were digested using 100 μg/mL of nuclease-free proteinase K (Invitrogen, Carlsbad, CA, USA). To detect EqPV-H DNA, the digoxigenin-labeled dsDNA probe that was denatured in the hybridization buffer (Enzo Life Sciences, Farmingdale, NY, USA) was applied to the tissues and hybridized at 45°C overnight. After hybridization, the slides were washed with a saline sodium citrate buffer (50 mM NaCl and 15 mM sodium citrate, pH 7.0). Then, the alkaline phosphate-conjugated anti-digoxigenin antibody was applied to the tissues. The ISH signal was visualized using nitro blue tetrazolium/5-bromo-4-chloro-3-indolyl phosphate. Finally, the slides were counterstained with 3% methyl green (Sigma, St. Louis, MO, USA) and covered with glass coverslips using VectaMount mounting medium (Vector Laboratories, Burlingame, CA, USA).

## 3. Results

### 3.1. Clinical Data and Physical Examination

A 14-year-old thoroughbred mare with a body weight of 500 kg was examined for inappetence, an increase in body temperature, and icterus on 12 June 2021. The mare was born in the USA and imported to Korea in 2017. According to the referring veterinarian and owner, the symptoms were recognized on the day before presentation and were found to be unimproved by symptomatic treatment, including fluid therapy, nonsteroidal anti-inflammatory drugs, and antibiotics. After entry to Korea in 2017, the mare received regular vaccination for equine influenza (Proteqflu, Merial), strangles (Equivac S, Zoetis), and Japanese encephalitis (Himmvac, KBNP). There was no history of administration of equine biological products known as sources of EqPV-H infection, although relevant information before 2017 was not available.

**Figure 1:**
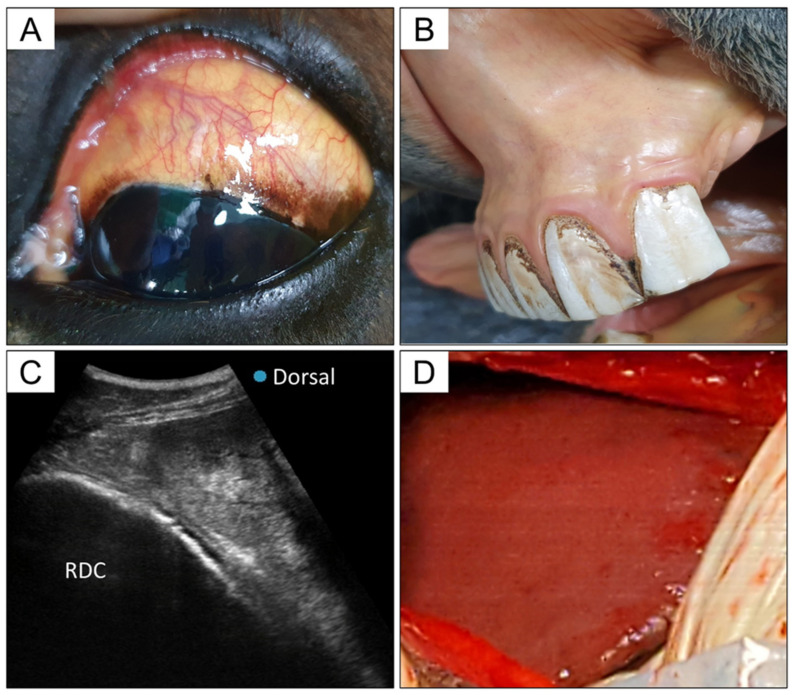
Clinical findings of Theiler’s disease case seen in this study. (**A**) Yellowish color change in the sclera. (**B**) Yellowish color change of mucous membrane. (**C**) Ultrasonographic image of the liver obtained from the right abdomen. The liver shows heterogeneous and irregular surface with a hyperechogenic structure in the liver parenchyma; RDC, right dorsal colon. (**D**) Liver appearance during laparotomy. The liver has remarkably blunt edges and irregular surfaces with dark-reddish color changes.

On presentation, the mare showed depressed mentation, hyperthermia (body temperature of 4 °C), decreased gut sound, 8–10% dehydration, and yellowish color of the sclera and mucous membranes (Figure 1A,B). Hematological investigation and biochemical analysis revealed polycythemia (hematocrit, 66.29%, reference range, 32–53%; red blood cell count, 12.9 × 10^12^/L, reference range, 6.8–12.9 × 10^12^/L), elevated quantities of liver-related enzymes (aspartate aminotransferase, 2366 U/L, reference range, 175–340 U/L; gamma-glutamyl transferase, 181 U/L, reference range, 5–24 U/L), increase in total bilirubin level (24.8 mg/dL, reference range, 0.5–2.3 mg/dL), and hyperlactatemia (5.4 mmol/L, reference range, 1–1.5 mM/L) (Table 1). The results are consistent with dehydration and liver failure. Abdominal ultrasonography (US) revealed a heterogeneous and irregular liver surface, with a hyperechogenic structure in the liver parenchyma (Figure 1C). Distinctly reduced intestinal motility was seen, and gas distention and displacement of the colon were identified on US and rectal examination.

To correct, and identify the cause of, hepatopathy and colic, an exploratory laparotomy was performed. We performed an abdominal midline incision under general anesthesia, and colonic gas and displacement were corrected. We observed some scattered petechial hemorrhages on the serous membrane of the small intestine. The liver had remarkably blunt edges and an irregular surface, with dark-reddish color changes (Figure 1D). After the laparotomy, the vitality and hyperlactatemia (5.4–3.3 mmol/L) were temporarily improved and the mare maintained normal vital signs. However, the clinical signs rapidly deteriorated and the mare was euthanized approximately 48 h after the detection of the first clinical sign.

### 3.2. Detection of EqPV-H DNA and Phylogenetic Tree Analysis

To identify the causative agents of hepatopathy, we determined the presence of three viruses using the conventional nested PCR technique: EqPV-H, EqHV, and HEV. Four samples (serum, oral swab, nasal swab, and liver biopsy tissue) were used for DNA detection, and EqPV-H DNA was positive in all four samples. Testing for the other viruses showed negative results. The results were confirmed through sequencing, and each EqPV-H amplicon sequence (391 bp without primer sequences) from all samples was identical to the others.

**Figure 2:**
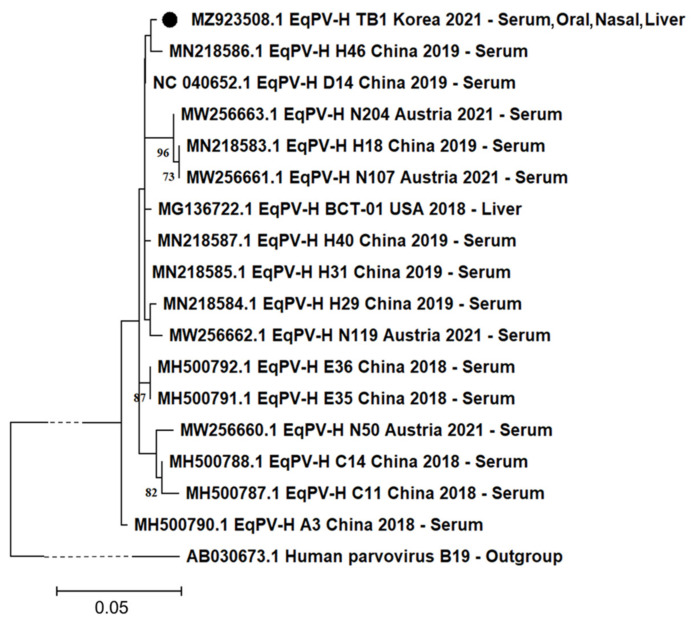
Phylogenetic analysis based on partial genomic sequence. A 391-nucleotide base pair segment encoding the partial NS1 protein from the Korean isolate (MZ923508.1, indicated with a black circle) was compared with the other corresponding sequences from the USA, China, and Austria. GenBank accession number, country, year of registration, and isolated tissue type are provided. The human parvovirus B19 (AB030673.1) was used as outgroup. For visualization purposes, the branch line of outgroup was indicated as dashed lines. Only bootstrap values of ≥70 were included.

The obtained partial NS1 gene of EqPV-H was genetically evaluated. In the phylogenetic tree analysis, the Korean isolate was most closely related to the Chinese strain H46 (Figure 2). We calculated the identity rate of the isolated gene with 16 other available EqPV-H NS1 sequences previously identified in the USA, China, and Austria. They showed a high similarity rate ranging from 97.95% to 99.49%. One unique nucleotide substitution (T1260C in NS1 CDS) was observed in the Korean isolate, compared to the other 16 viruses. Moreover, the Korean isolate had the same unique nucleotide substitution (T1222C in NS1 CDS) as that reported in Chinese strains, compared to the USA strain BCT-01 [19].

### 3.3. Histopathology and In Situ Hybridization

**Figure 3:**
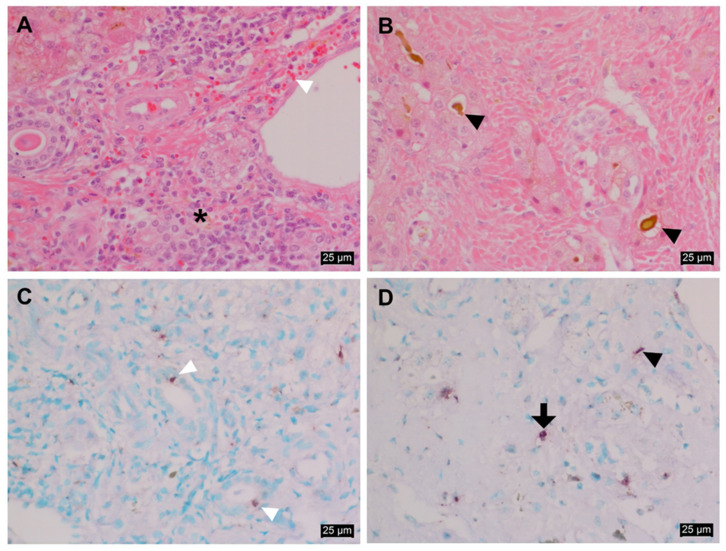
Histopathologic observation and in situ hybridization of EqPV-H in the liver tissues. (**A**) Moderate lymphoplasmacytic hepatitis (indicated with an asterisk) with mild hemorrhage (indicated with a white arrowhead). (**B**) Severe necrosis with cholestasis (indicated with a black arrowhead). The cells were stained using a hematoxylin and eosin (H&E) stain. Magnification: ×400. (**C**) The individual epithelial cells of the bile duct appearing positive for EqPV-H DNA (indicated with a white arrowhead). (**D**) Hepatocytes (indicated with a black arrow) and Kupffer cells (indicated with a black arrowhead) were positive for EqPV-H DNA. Methyl green counterstain. Magnification: ×400.

The histopathology of the liver infected with EqPV-H is shown in Figure 3A,B. Moderate to severe necrotizing lymphoplasmacytic hepatitis was prominently observed in this case. Hepatic cells were found to be severely swollen. Moderate lymphocytic/plasmacytic inflammatory cells were found to be infiltrated in the periportal area (Figure 3A). In particular, the deposition of bile juice was present in the severely necrotizing lesion and the cytoplasm of some hepatocytes (Figure 3B).

In situ hybridization findings demonstrated strong positive signals for EqPV-H DNA in the infected cell types, which were determined by a pathologist based on their morphology-related features (Figure 3C,D). EqPV-H DNA was found in the cytoplasm of the bile duct epithelium, hepatocytes, and Kupffer cells. In particular, some individual epithelial cells of the bile duct in the periportal area were positive for the DNA (Figure 3C). The cytoplasm and nuclei of hepatocytes and Kupffer cells were positive for EqPV-H DNA near the necrotic lesion (Figure 3D). A PCR-negative liver tissue sample from another normal horse was used as a negative control (Supplementary Figure S1).

## 4. Discussion

In this paper, we report the first clinical case of EqPV-H-associated TD with fulminant liver failure and we identify the genetic character of the isolated virus DNA.

TD was first described in 1918, but the etiology of TD remained unclear until the discovery of EqPV-H in 2018; currently, EqPV-H is considered as a major causative agent in TD [2]. Despite the widespread distribution and high prevalence of EqPV-H, clinical investigation remains in its infancy and the risk of EqPV-H infection is underestimated [2,11,15,16,17,18,19]. In Asia, two EqPV-H epidemiological surveillance studies were recently conducted in China, but no clinical case has yet been reported [2,16,19].

The clinical findings reported in this study, such as depression, icterus, pyrexia, and changes in blood parameters, are consistent with typical hepatic failure and with previously reported TD cases [2,5,6,11,27,28]. In addition to physical examination findings, abdominal US findings support the diagnosis of hepatopathy. However, neurological signs, including hepatic and metabolic changes, are not uncommon in colic patients [29,30]. These similarities may create confusion among practitioners and could lead to incorrect diagnosis and treatment. Thus, practitioners are encouraged to include EqPV-H infection in the differential diagnosis of colic and hepatic failure.

In this study, three viruses implicated in hepatitis were tested using serum, nasal swab, oral swab, and liver biopsy samples. EqPV-H DNA was detected in all four specimens, while the other viruses showed negative results. We present the first field evidence for nasal and oral shedding of EqPV-H. A previous experimental study showed nasal and oral shedding of EqPV-H during the viremia period, as well as horizontal transmission through oral inoculation [3]. Consequently, natural viral shedding and viral accumulation in the environment will increase the risk of virus transmission via the oral route. Our results suggest the occurrence of oral viral shedding, which could lead to the horizontal transmission of EqPV-H via the oral route. However, natural shedding of EqPV-H DNA does not imply viral infectivity, and additional data and studies are needed to determine the correlation between viral shedding and horizontal transmission.

Recently, a study of the clinical course of EqPV-H described persistent infection of EqPV-H over five years that resulted in chronic viremia in subclinical horses [14]. Thus, it is plausible that the horse in our case may have been in a subclinical condition, showing chronic viremia, for some time. The detection of anti-EqPV-H antibodies could provide information about whether the horse had been infected prior to admission. However, we could not test for anti-EqPV-H antibodies because there are still few options for antibody detection in the field. In some of the previous papers dealing with EqPV-H [11,14], specific antibodies were measured using luciferase immunoprecipitation systems. However, there are no commercial kits for field testing. This is a limitation of our study.

The phylogenetical tree of the isolated partial NS1 region revealed that the virus is closely related to the Chinese strain (Figure 2). In previous data, genetic diversity among EqPV-H variants is generally low, but distinct geographical patterns are observed [2,19]. The Korean isolate (391 base pair size) showed a close relationship with the Chinese strain in the phylogenetic tree and shared the same unique nucleotide variation, which was distinct from the USA strain BCT-01. At the same time, the Korean isolate also showed its own trait, having another unique nucleotide variation different from the other 16 available strains worldwide in the NS1 gene of EqPV-H. These results indicate that further studies are needed to enable the genetic characterization of Korean EqPV-H strains and the identification of the effect of variation on the pathogenicity.

Histopathological analysis of the liver revealed severe necrosis in the liver, together with cholestasis and lymphoplasmacytic hepatitis. The ISH results confirmed the intralesional presence of EqPV-H DNA. Notably, EqPV-H DNA was found not only in the hepatocytes but also in the epithelial cells of the bile duct and in Kupffer cells. In the previous experimental study, EqPV-H nucleotides were detected via the qPCR technique in various organ tissues, including the liver [3]; however, the specific cell types in liver relating to EqPV-H infection and tropism remain unknown. Our ISH results directly show the ability of EqPV-H to infect a wide range of cells in addition to hepatocytes. Moreover, this could be a clue to rationalizing the hepatotropism of EqPV-H, which is not common in other parvovirus species because most parvoviruses require actively dividing cells for their replication [4]. Further investigations are needed on this topic to validate the results.

## 5. Conclusions

In conclusion, we report the first clinical case of EqPV-H-related TD in Asia, which occurred in Korea. Together with specific descriptions of the clinical course of the infection, we provide several new discoveries regarding viral shedding of EqPV-H, its genetic characteristics, and the cell infectivity in the liver. Our clinical case will expand both the clinical and experimental bases for understanding and preventing EqPV-H-related hepatitis. Our data will benefit practitioners and researchers by contributing to a better understanding of EqPV-H. However, further studies are warranted regarding worldwide epidemiology, pathogenicity, and clinical relevance as an important risk factor for the horse industry.

## Figures and Tables

**Table 1:** Hematological and serum biochemical parameters observed in this EqPV-H + case.

Parameters.	Day 1	Day 2	Reference Range
AST	2366 U/L	NA	175–340 U/L
GGT	181 U/L	NA	5–24 U/L
TBIL	24.8 mg/dL	NA	0.5–2.3 mg/dL
Lactate	5.4 mmol/L	3.3 mmol/L	1–1.5 mM/L
Hematocrit	66.29%	77.25%	32–53%
Red blood cell count	12.9 × 10^12^/L	15.02 × 10^12^/L	6.8–12.9 × 10^12^/L
Neutrophil	10.67 × 10^9^/L	16.87 × 10^9^/L	2.3–9.5 × 10^9^/L
Lymphocyte	2.36 × 10^9^/L	0.91 × 10^9^/L	1.5–7.7 × 10^9^/L

Abbreviations: AST, aspartate aminotransferase; GGT, gamma-glutamyl transferase; TBIL, total bilirubin; NA, not available.

## Data Availability

The sequence reported in this study have been deposited in the GenBank database (accession no. MZ923508).

## Supplementary Materials

The following are available online at https://www.mdpi.com/article/10.3390/v13101917/s1, Table S1: Primer sets and PCR information obtained in this study, Figure S1: The H&E and ISH of normal equine liver.
