# Supplementary material for: First Clinical Case of Equine Parvovirus-Hepatitis-Related Theiler’s Disease in Asia
Source: Viruses. 2021 Sep 24;13(10):1917. doi: 10.3390/v13101917 (PMC8541225; doi:10.3390/v13101917)
Supplement: Supplementary file 1 [file viruses-13-01917-s001.zip › viruses-1388809-supplementary.pdf]

viruses

MDPI

Supplementary Materials

First Clinical Case of Equine Parvovirus-Hepatitis-Related Theiler's Disease in Asia

Table S1. Primer sets and PCR information obtained in this study.

PCR	Primer Set	Sequence (5′→3′)	Product Size [a]	T_m	PCR Cycle	Reference
EqPV-H detection	External forward	GGAGAAGAGCGCAACAAATGCA	454 bp	53 °C	35 cycles	[11]
	External reverse	AAGACATTTCCGGCCGTGAC-3				
	Internal forward	GCGCAACAAATGCAGCGGTTCGA	435 bp	57 °C	35 cycles	
	Internal reverse	GGCCGTGACGACGGTGATATC				
EqHV detection	External forward	TCCACCTATGGTAAGTTCTTAGC	451 bp	52 °C	35 cycles	[22,23]
	External reverse	ACCCTGTCATAAGGGCGTC				
	Internal forward	ACGGGGCAGARTCYAAAGGYGTW	265 bp	55 °C	35 cycles	
	Internal reverse	TCCAARCCCCGATAGTARGTGAC				
HEV detection	External forward	CAAGGHTGGCGYTCKGTTGAGAC	506 bp	55 °C	35 cycles	[24]
	External reverse	CCCTTRTCCTGCTGAGCRTTCTC				
	Internal forward 1	GYTCKGTTGAGACCACGGGYG	458 bp	55 °C	35 cycles	
	Internal forward 2	GYTCKGTTGAGACCTCTGGTGT				
	Internal reverse	TTMACWGTCRGCTCGCCATTGGC				

Abbreviations: PCR, polymerase chain reaction; T_m, melting temperature; bp, base pair; HEV, hepatitis E virus; EqHV, equine hepacivirus; EqPV-H, equine parvovirus-hepatitis.

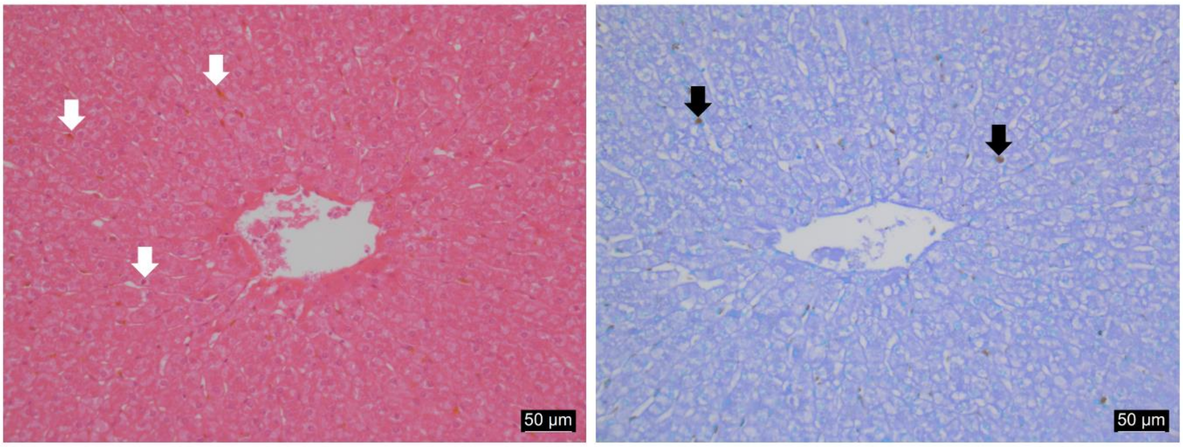

Figure S1. The H&E and ISH of normal equine liver. Equine parvovirus was not detected in normal liver tissues. Dark spots (black arrows) in ISH are formalin pigments. These formalin pigments were confirmed as brownish dots (white arrows) in H&E slide. These non-specific pigments were not observed in parvovirus-infected liver.
